# Adaptive prospective ECG-triggered sequence coronary angiography in dual-source CT without heart rate control: Image quality and diagnostic performance

**DOI:** 10.3892/etm.2012.828

**Published:** 2012-11-23

**Authors:** CHANG-JIE PAN, NONG QIAN, TAO WANG, XIAO-QIANG TANG, YUE-JUN XUE

**Affiliations:** Department of Radiology, The Second People’s Hospital of Changzhou, Nanjing Medical University, Changzhou, Jiangsu 213003, P.R. China

**Keywords:** dual-source computed tomography, non-invasive coronary angiography, coronary angiography, heart rate

## Abstract

The aim of this study was to evaluate the accuracy of using second generation dual-source CT (DSCT) to obtain high quality images and diagnostic performance and to reduce the radiation dose in adaptive prospective electrocardiography (ECG)-triggered sequence (CorAdSeq) CT coronary angiography (CTCA) without heart rate control. No prescan β-blockers were administered. Un-enhanced CT and CTCA with adaptive prospective CorAdSeq scanning without heart rate control were performed in 683 consecutive patients divided into two body mass index (BMI) groups: BMI <25 kg/m^2^ (group A, n=412) and BMI ≥25 kg/m^2^ (group B, n=271). The image quality and quantitative stenosis of all coronary segments with a diameter ≥1 mm were assessed. The mean heart rate (MHR), heart rate variability (HRV) and radiation dose values were recorded. In 426 cases, the diagnostic performance was evaluated using quantitative conventional coronary angiography as the reference standard. Diagnostic image quality was obtained in 98.5% of segments in group A and in 98.8% of segments in group B, with no significant differences between the groups. No correlations were observed between the image quality score and MHR or HRV (P=0.492, P=0.564, respectively). The effective radiation doses in groups A and B were 2.57±1.01 mSv and 6.36±1.88 mSv, respectively. The sensitivities and specificities of diagnosing coronary heart disease per patient were 99.6% and 97.8% in group A and 99.5% and 97.5% in group B, respectively (P>0.05). Adaptive prospective CorAdSeq scanning, without heart rate control, by second generation DSCT had a high image quality and diagnostic performance for coronary artery stenosis with lower radiation doses.

## Introduction

Non-invasive CT coronary angiography (CTCA) has evolved into an important clinical tool for the assessment of coronary artery disease (CAD) (1). According to guidelines, its use is considered appropriate for symptomatic patients with an intermediate risk of CAD (2). Specifically, the wide-spread use of 64-slice CT and dual-source CT (DSCT) in coronary angiography has greatly improved the image quality and accuracy of diagnosis (3), although the radiation dose has also been increased (4). In a recent multicenter, multivendor trial, Hausleiter *et al*(5) demonstrated a median effective radiation dose of 12 mSv for CTCA with retrospective electrocardiography (ECG) gating, while certain sites used protocols exceeding even 30 mSv.

Furthermore, adaptive ECG-pulsing algorithms for use in spiral CT are designed to maintain diagnostic image quality in arrhythmic patients since the continuous high X-ray tube output allows flexible selection of the desired reconstruction phase throughout the R-R interval. However, image quality is only maintained at the cost of higher radiation exposure (6). Due to the inevitable problem of high radiation doses in CT examination, various strategies to reduce the radiation exposure of patients have been developed. The most significant is prospectively ECG-gated CTCA, also called step-and-shoot (SAS) mode. Low radiation doses ranging between 1.2 and 4.3 mSv have been reported using various 64-slice and first-generation, dual-source 64-slice CT (7,8). Most significantly, this low-dose SAS method provides high image quality (8,9), although it remains necessary to control the heart rate.

The purpose of the present study was to evaluate the accuracy of using second generation DSCT to obtain high quality images and diagnostic performance and to reduce the radiation dose in adaptive prospective ECG-triggered sequence (CorAdSeq) CTCA without heart rate control.

## Materials and methods

### Patient population

**Figure 1.**
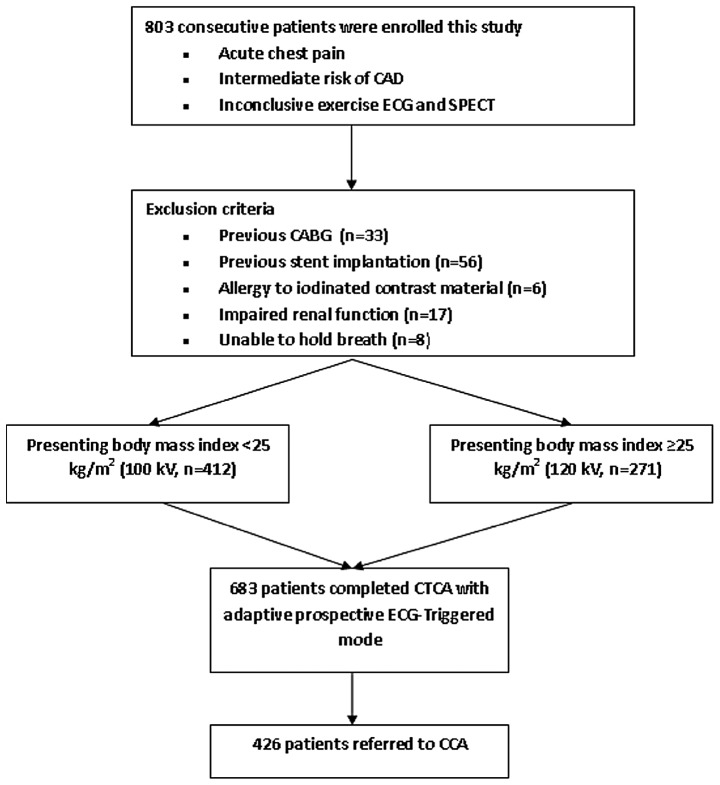
Flowchart of study patients. CAD, coronary artery disease; CABG, coronary artery bypass graft; CTCA, CT coronary angiography, ECG, electrocardiography; CCA, conventional coronary angiography; SPECT, single-photon emission computed tomography.

Between June 2010 and June 2011, 803 consecutive symptomatic patients suspected of having or with known CAD were eligible for inclusion in the present study (Fig. 1). Those patients with previous coronary artery bypass grafts (n=33) and stent implantation (n=56) were excluded. The exclusion criteria specific to CTCA were known allergies to iodinated contrast material (n=6), impaired renal function (serum creatinine levels >120 *μ*mol/l; n=17) and the inability to hold a breath (n=8). Thus, the study population consisted of 683 patients (447 males, 236 females; mean age ± standard deviation, 61.2±12.0 years). This study was conducted in accordance with the declaration of Helsinki and with approval from the Ethics Committee of Nanjing Medical University (Tianning, Changzhou, Jiangsu, China). Written informed consent was obtained from all participants.

### Scan preparation

No β-blockers or other drugs were used to decrease heart rate or/and change arrhythmia prior to scanning. An 18-G needle was embedded in the middle vein of the right elbow. The skin was cleaned and ECG lines were placed in standard positions. Prior to scanning, the patients received one sublingual dose of nitroglycerin aerosol spray (Jingwei Pharma Co., Ltd., Shandong, China) to expand the coronary artery.

### Scan protocol and image post-processing

All patients were scanned on a second-generation DSCT scanner (Somatom Definition Flash; Siemens Medical Solutions, Munich, Germany) with the adaptive prospective CorAdSeq model. The imaging parameters were as follows: detector collimation, 2×64×0.6 mm; slice acquisition, 2×128×0.6 mm by means of a z-flying focal spot; gantry rotation time, 280 msec. The tube voltage was adjusted to each patient’s body mass index (BMI); 100 kV was used for a BMI <25.0 kg/m^2^ and 120 kV for a BMI of ≥25.0 kg/m^2^ for each technique. Automatic exposure control system-based tube current modulation (Care-DOSE; Siemens Medical Solutions) was used in all patients with the two data acquisition techniques. The reference tube current was 390 mA, with the tube current automatically adjusted to the size and density of the body region (10) and a full tube current applied between 30–80% of the R-R interval, so as to obtain both systolic and diastolic images at full dose. A scout view of the thorax was obtained to plan CT angiography data acquisition and the scanning scope was performed from 2 cm below the level of the tracheal bifurcation to the diaphragm in a cranio-caudal direction. All examinations were performed following a verbal command, instructing the patient to hold their breath after a deep inspiration. The breath-hold maneuver was practiced once prior to the actual examination. Patients intravenously received 70 ml (for BMI <25.0 kg/m^2^) or 80 ml (for BMI ≥25.0 kg/m^2^) non-ionic contrast medium (Ultravist 370 mgI/ml; Bayer Schering Pharma, Berlin, Germany) at a flow rate of 4.5 ml/sec (for BMI <25.0 kg/m^2^) or 5.0 ml/sec (for BMI ≥25.0 kg/m^2^), adjusted to each patient’s BMI respectively, followed by a saline chaser of 40 ml at the same flow rate, using a binocular high-pressure injector. Data acquisition was started 4 sec after a region of interest in the ascending aorta reached a threshold of 150 HU (bolus tracking technique). The data were rebuilt and transferred automatically to the workstation (Syngo MMWP VE36A, Siemens Medical Solutions).

Images for the two protocols were reconstructed with a slice thickness of 0.75 mm, a reconstruction increment of 0.5 mm and using a soft-tissue convolution kernel (B26f). For vessel wall calcification, additional images were reconstructed using a sharp-tissue convolution kernel (B46f) to compensate for blooming artefacts. The post-processing included maximum intensity projection (MIP), multiplanar reformation (MPR) and volume rendering (VR).

### Assessment of image quality

Image quality was evaluated in a double-blind manner and scored by two experienced radiologists (T.W. and X.Q.T., each with ≥3 years experience of interventional cardiology) who identified all available coronary segments in invasive coronary angiography using the 17-segment modified American Heart Association classification (11). Of the 683 patients, 426 (62.4%, 426/683) underwent conventional coronary angiography (CCA). All conventional angiograms were performed within one month before or after CTCA. All segments with diameters ≥1 mm were included for comparison with CTCA. The stenoses were classified as significant if the lumen diameter reduction was ≥50%.

The image quality assessment criteria used a semi-quantitative five-point grading scale (12), as follows: 5 points, images had clear coronary edge and no motion artifacts; 4 points, images had slightly blurred edges and only mild motion artifacts; 3 points, images had moderately blurred edges and mild motion artifacts without significant splitting, not impairing the diagnosis; 2 points, images with blurred edges and clear motion artifacts; and 1 point, images with the coronary lumen unidentifiable and thus undiagnosable. Images of ≥3 points were regarded as diagnostic image quality.

The mean heart rate (MHR) and minimal and maximal heart rate per minute were recorded following CT acquisition for each patient. Heart rate variability (HRV) was calculated as the maximum bpm - minimum bpm.

### Radiation dose assessment

The volume CT dose index (CTDIvol, in mGy) and dose length product (DLP, in mGy x cm) were automatically generated by the machine and recorded for each patient during examination. Effective dose (ED, in units of mSv) was calculated using the formula ED = DLP × C (13), where C is the conversion factor (C=0.01 4 mSv × mGy^−1^ × cm^−1^) (14). CTDIvol and ED were presented as mean values ± standard deviation.

### Statistical analysis

The patients and scan characteristics were expressed as numbers and percentages, while continuous variables were expressed as mean values ± standard deviation. Statistical analyses were performed using statistical software (SPSS, v.13.0 for Windows; SPSS, Chicago, IL, USA). The diagnostic performance of CTCA for the diagnosis of significant CAD compared with the reference standard, quantitative coronary angiography, at CCA, was determined using sensitivity, specificity, positive predictive value (PPV) and negative predictive value (NPV) and their corresponding 95% confidence intervals. The differences in patients and scan characteristics were calculated using the Student’s t-test. The image quality according to the MHR and HRV groups was compared using Fisher’s exact test. An α level <0.05 was considered to indicate a statistically significant difference. For the dose estimates, the two-way analysis of variance test was performed to evaluate the effect of MHR and HRV on the radiation exposure (CTDIvol and ED), for each BMI group. An α level <0.05 was considered to indicate a statistically significant difference. When there were differences between groups, the multi-comparison correction was used to adjust the α level by the Bonferroni method. The interobserver agreement on semi-quantitative grades of image quality between the two readers was calculated prior to consensus reading by using κ statistics. A κ value >0.81 corresponded to excellent interobserver agreement, with values of 0.61–0.80 corresponding to good agreement (15).

## Results

### Patient demographics

**Figure 2.**
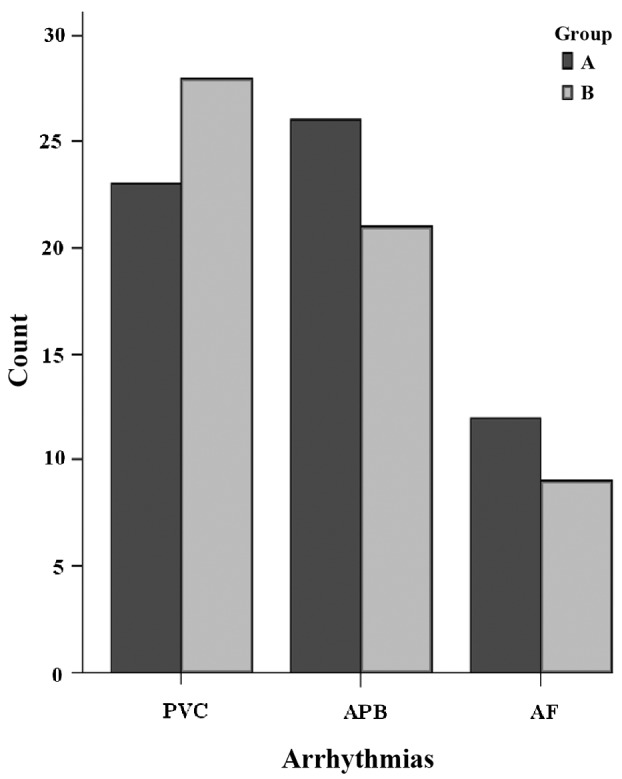
Graph showing the cases of arrhythmia in the two groups. PVC, premature ventricular contraction; APB, atrial premature beat; AF, atrial fibrillation.

The comparisons of demographic data for the patient groups are listed in Table I. No significant differences were observed in any of the demographic parameters between the two groups. A total of 683 patients were eligible for CTCA (group A, BMI <25 kg/m^2^, n=412 and group B, BMI ≥25 kg/m^2^, n=271). Overall, 356 patients had a heart rate >70 bpm during scanning (52.1% of 683 patients), with 195 in group A (47.3%, 195/412) and 161 in group B (59.4%, 161/271). Of the 683 patients, 54 had a heart rate >100 bpm (7.9%, 54/683), with 24 in group A (5.8%, 24/412) and 30 in group B (11.1%, 30/271). The MHR was 78.5±13.2 bpm in group A and 79.0±13.9 bpm in group B (P=0.711) and the HRV was 20.7±19.3 bpm in group A and 23.7±26.8 bpm in group B (P= 0.201). No significant differences were observed between the two groups. Of the patients, 564 (82.6%, 564/683) remained in sinus rhythm during data acquisition and 119 (17.4%, 119/683) exhibited irregular heart beats, including premature ventricular contraction (n=51), premature atrial beat (n=47) and atrial fibrillation (n=21). No significant differences were identified between the two groups (P=0.78; Fig. 2).

### CT image quality

A five-point rating scale was used to assess the image quality. A total of 683 coronary arteries were included in the present study, of which 671/683 (98.2%) yielded high image quality and 12/683 (1.8%) showed non-diagnostic image quality due to severe respiratory motion. No coronary segment was considered non-diagnostic due to elevated MHR or HRV. A total of 98.5% (6493/6592) of the segments in group A and 98.8% (4286/4336) in group B were evaluated. The interobserver agreement for image quality ratings between the readers was excellent (κ=0.856). No significant differences were observed between the number of segments depicted with diagnostic image quality in the two groups (P=0.158). Images with poor diagnostic quality were caused by artifacts associated with respiratory motion (n=7; group A, 4; group B, 3) and associated with severe coronary calcifications (n=5; group A, 2; group B, 3). The mean image quality scores for groups A and B were 4.77±0.46 and 4.83±0.37, respectively. No significant differences were identified between the two groups (P=0.133). No correlation was observed between the image quality score and the MHR or HRV (P=0.492, P=0.564, respectively).

### Radiation dose assessment

The CTDIvol, DLP and ED estimates were all significantly lower for group A than for group B (P<0.001, P<0.05, correction, Table I), while the scan range was not significantly different between the two groups (P=0.228; Table I). In patients with a BMI <25.0 kg/m^2^, the estimated ED was 2.57±1.01 mSv and when the BMI was ≥25.0 kg/m^2^, the ED was 6.36±1.88 mSv.

The MHR and HRV with ED and CTDIvol had a significant (P<0.01, P<0.05, respectively) negative correlations between the two groups, which may be attributed to the increase in pitch values.

### Diagnostic performance

**Figure 3.**
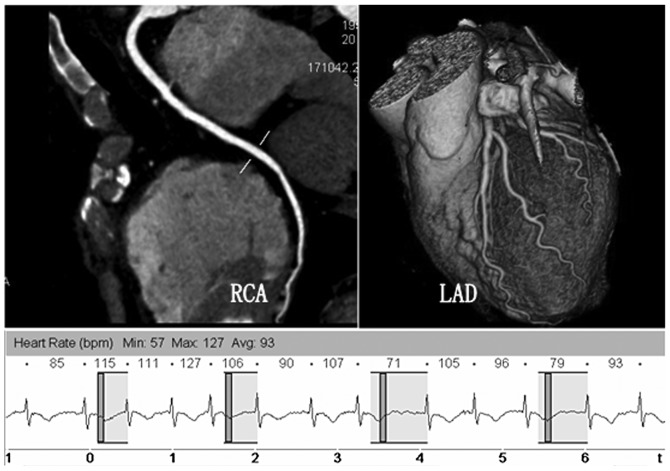
Female, 58-year-old patient, BMI=20.69 kg/m^2^. The HRV was 44 bpm during scanning. MHR and heart rate range were 93 and 57–127 bpm, respectively. Image quality score was 5 points. CT coronary angiogram (left) and volume-rendered reconstruction (right) show high quality images of the RCA and LAD. BMI, body mass index; HRV, heart rate variability; MHR, mean heart rate; RCA, right coronary artery; LAD, left anterior artery.

CCA was used as the reference standard and demonstrated the prevalence of CAD in 469 patients to be 68.7%. The prevalence was 68.4% in group A (282/412) and 69.0% in group B (187/271). In these 469 patients, 571 coronary stenoses of ≥50% lumen diameter reduction were detected by CCA [15 in the left main, 285 in the left anterior descending (Fig. 3), 189 in the left circumflex and 82 in the right coronary artery]. Of the 562 coronary stenoses of ≥50% lumen diameter reduction that were shown by DSCT, 9.1% (593/6493) significant stenosis per segment was observed in group A and 9.7% (415/4286) in group B. No significant differences were observed between the proportion of segments depicted with significant stenoses per segment between the two groups (P=0.344). Of the coronary segments, 68 were classified as false-positives by DSCT: 44 were unevaluable and thus estimated as having significant stenosis, but did not exhibit significant stenosis on CCA and 24 segments were evaluated as false-positives since the degree of lumen reduction was overestimated. Thus, the κ value for coronary artery stenosis detection with CTCA was 0.985, indicating high intermethod agreement between readers. The A and B group data for the segment-based, vessel-based and patient-based sensitivity, specificity, PPV and NPV are presented in Table II.

## Discussion

**Figure 4.**
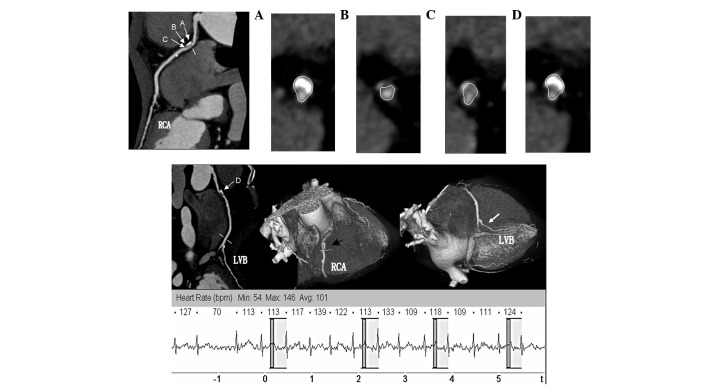
Male, 65-year-old patient with atrial fibrillation, BMI=22.54 kg/m^2^. HRV was 11 bpm during scanning; MHR and heart rate range were 101 and 54–146 bpm, respectively. Image quality score was 5 points. CT coronary angiograms show cross-sections (A–D) of (upper left) proximal RCA and (lower left) LVB. Volume-rendered reconstructions (lower middle and right) showing >50% stenosis (arrows). BMI, body mass index; HRV, heart rate variability; MHR, mean heart rate; RCA, right-coronary artery; LAD, left-anterior artery.

With the growing popularity of 64-Multidetector CT (MDCT) and DSCT, CTCA has gained a wide spectrum of applications due to its simplicity, non-invasiveness and high accuracy in detecting significant stenoses in patients with regular and low (<65 bpm) heart rates. β-blockers are commonly administered prior to CTCA to lower the heart rate, thereby reducing the number of image-degrading motion artifacts. DSCT scanners provide an improved temporal resolution compared with single-source CT equipment and may eliminate the need for prescan β-blockers (16). However, in previous studies, the number of patients with increased heart rate (>80 bpm) was small and patients with arrhythmias were excluded from the majority of studies (3,16). Heart rate modulation by oral or intravenous administration of β-blockers was not required prior to the scanning procedure in the present study. The present technique of the CorAdSeq demonstrated high image quality, even in a number of patients with arrhythmias (Fig. 4). No correlation was identified between the image quality and MHR or HRV.

A small number of studies have investigated the effect of HRV on image quality and diagnostic performance. However, in these studies the HRVs were defined as the standard deviation of the MHR during CTCA. A sudden change in heart rate may cause several problems in the acquisition of CTCA since artifacts are created due to differences in the image reconstruction phases between consecutive heart beats. Previous studies did not perform CTCA in patients with arrhythmias. In the present study, 7.9% (54/683) patients had a heart rate >100 bpm during scanning and 119 patients (17.4%) had arrhythmias. Diagnostic image quality was obtained for all patients on the basis of adaptive prospective CorAdSeq, even patients with severe HRV. This finding indicates that CorAdSeq is a robust technique and may be used in all patients undergoing CTCA.

DSCT uses two mutually perpendicular tubes, one rotation of which significantly increases the covered area and scan speed. Using a 0.33 sec gantry rotation time, the time resolution is one quarter (1/4) of the rotation time, i.e. 83 msec (17). In the present study group, the gantry rotation time was reduced to 0.28 sec and the time resolution was increased to 75 msec. Such a time resolution was enough for coronary CT scans without the need to control heart rates. By contrast, conventional perspective CorAdSeq scanning requires more strict control of heart rate and rhythm (heart rate <70 bpm) and heart rate fluctuation <10 times/min (3,12). Therefore, the conventional technique may only be applied to patients with a regular rhythm and low heart rate. Furthermore, the time phase cannot be changed when restructuring and split images are likely to appear if the heart rate change is abrupt, thereby affecting the image quality. CorAdSeq scanning selects appropriate reconstruction phases to control the exposure to X-rays using the heart rate monitoring by ECG. This has the advantage of increasing the scanning speed and lowering radiation dose as the X-ray exposure only occurs in selected phases rather than in the whole cardiac cycle. If arrhythmia occurs, the patient table stops in the original position without scanning and data collection. Only when the next R-R wave becomes rhythmic is the patient table moved to the appropriate position and scanning and data acquisition restarted. Since the examination bed does not move, there is no data gap in the cardiac cycle before and after arrhythmia. A high overall diagnostic performance was observed for DS CTCA in the detection of significant coronary artery stenosis with a sensitivity of 99% and an NPV of 99% on a per-patient basis. These results were obtained without excluding any segments or patients on the basis of non-diagnostic image quality. No significant differences were observed in image quality between the two groups regardless of MHR and HRV. Despite high heart rates (maximal 171 bpm and MHR 78.8±13.6 bpm) and large heart rate fluctuations (22.2±23.4 bpm) during scanning in the patients, 98.2% (671/683) yielded high quality images. No coronary segments were considered non-diagnostic due to elevated MHR or HRV, indicating that the image quality obtained by CorAdSeq scanning was not significantly affected by increased heart rate or arrhythmia. In the present study, the overall average image score for all the patients was 4.80±0.41.

Numerous factors affect CTCA radiation dose, including scan length, scan speed and tube voltage (18). The biggest difference between CorAdSeq and DSCT retrospective ECG-gated spiral scanning is CordAdSeq’s CARE Dose 4D technique. CARE Dose 4D is based on the approach of modulating the tube current and keeping the image noise constant from patient to patient and over the whole scan. The reference current is adjusted automatically according to the anatomy of the patient’s body and organs. In slim patients, the CARE Dose 4D automatically reduces the current in the CTCA scanning. In obese patients, it increases the current when CTCA scanning and so is able to further reduce the radiation dose. SAS CTCA has attracted interest as a technique for reducing radiation exposure while preserving diagnostic image quality. However, SAS CTCA is currently limited to selected patients with low and regular heart rates only (9). The present findings confirm and corroborate those of previous reports (7,9,12) investigating prospective ECG gating and demonstrate the clinical feasibility of the technique as an effective method for reducing radiation exposure without affecting image quality. It should, however, be emphasized that these results were obtained in a selected groups of patients. If the prospective gating technique is applied to patients with low (<70 bpm) and regular heart rates, that would only demonstrate that this technology is applicable to such patients. More significantly, the radiation dose of such a method remains higher. Under the same conditions, in the present study of patients with a BMI <25.0 kg/m^2^, the ED of was 2.57 mSv±1.01, while for those with a BMI ≥25.0 kg/m^2^, the ED was 6.36 mSv±1.88, although the average DLP and ED were slightly lower than in relevant studies (10,11) and significantly reduced compared with retrospective ECG-gated scanning or 64-MDCT (19,20).

The present study had certain limitations. Firstly, the classification of the subgroups was arbitrary and was not categorized according to MHR and HRV. Secondly, evaluation was not performed between the image quality and diagnostic accuracy. The association between the two should be the subject of further studies.

In conclusion, using CTCA with the new generation DSCT adaptive perspective CorAdSeq is feasible in patients without heart rate control. This greatly widens the scope of its applications with increased image quality and reduced radiation exposure in coronary artery imaging.

## Figures and Tables

**Table I.** Patient demographics and scan parameters.

Factors	Group A (n=412)	Group B (n=271)	P-value
Patient characteristic			
Gender (male/female)	268/144	179/92	0.787
Age (years)^a^	61.8±12.3	60.6±11.7	0.327
Body weight (kg)^a^	67.6±6.9	73.4±9.3	0122
BMI (kg/m^2^)^a^	23.47±0.97	26.10±0.75	0.000
Scan parameter			
MHR (bpm)^a^	78.5±13.2	79.0±13.9	0.711
HRV^a^	20.7±19.3	23.7±26.8	0.201
Scan range (mm)^a^	115±13	117±11	0.228
Scan duration (s)^a^	5.8±0.8	6.2±0.6	0.212
CTDIvol (mGy)^a^	16.05±3.84	38.64±11.63	0.000
DLP (mGy × cm)^a^	183.89±56.99	454.18±166.26	0.000
ED (mSv)^a^	2.57±1.01	6.36±1.88	0.000
Score	4.77±0.46	4.83±0.37	0.133

Note:

a Data are the mean ± standard deviation. The tube voltage was 100 kV for patients with a BMI of <25 kg/m^2^ (group A) and 120 kV for those with a BMI of ≥25 kg/m^2^ (group B). BMI, body mass index; MHR, mean heart rate; HRV, heart rate variability; CTDIvol, volume CT dose index; DLP, dose length product; ED, effective dose.

**Table II.** Comparison of the two groups by CT coronary angiography for the diagnosis of significant coronary stenosis.

	Group A (BMI<25 kg/m^2^)			Group B (BMI≥25 kg/m^2^)		
	Segment-based	Coronary-based	Patient-based	Segment-based	Coronary-based	Patient-based
TP	587	369	272	410	198	189
FP	38	4	3	30	5	2
FN	6	2	1	5	1	1
TN	5862	1270	136	3841	876	79
Sensitivity (%)	99.0 (98.2–99.8)	99.5 (98.8–100)	99.6 (98.9–100)	98.8 (97.8–99.8)	99.5 (98.5–100)	99.5 (98.5–100)
Specificity (%)	99.4 (99.2–99.6)	99.7 (99.4–99.9)	97.8 (95.4–100)	99.2 (98.9–99.5)	99.4 (98.9–99.9)	97.5 (94.1–100)
PPV (%)	93.9 (92.0–95.8)	98.9 (97.0–99.9)	98.9 (97.7–100)	93.2 (90.9–95.6)	97.5 (95.4–99.6)	98.9 (97.6–100)
NPV (%)	99.9 (99.8–100)	99.8 (99.6–100)	99.3 (97.9–100)	99.9 (99.8–100)	98.9 (99.7–100)	98.8 (96.4–100)

Numbers in parentheses are 95% confidence intervals. BMI, body mass index; FN, false negatives; FP, false positives; NPV, negative predictive value; PPV, positive predictive value; TN, true negatives; TP, true positives.
